# Deep Q-network to produce polarization-independent perfect solar absorbers: a statistical report

**DOI:** 10.1186/s40580-020-00233-8

**Published:** 2020-08-03

**Authors:** Iman Sajedian, Trevon Badloe, Heon Lee, Junsuk Rho

**Affiliations:** 1 Department of Materials Science and Engineering, Korea University, Seoul, 02842 Republic of Korea; 2 Department of Mechanical Engineering, Pohang University of Science and Technology (POSTECH), Pohang, 37673 Republic of Korea; 3 Department of Chemical Engineering, Pohang University of Science and Technology (POSTECH), Pohang, 37673 Republic of Korea

**Keywords:** Reinforcement learning, Deep Q-learning, Perfect solar absorbers, Statistical analysis

## Abstract

Using reinforcement learning, a deep Q-network was used to design polarization-independent, perfect solar absorbers. The deep Q-network selected the geometrical properties and materials of a symmetric three-layer metamaterial made up of circular rods on top of two films. The combination of all the possible permutations gives around 500 billion possible designs. In around 30,000 steps, the deep Q-network was able to produce 1250 structures that have an integrated absorption of higher than 90% in the visible region, with a maximum of 97.6% and an integrated absorption of less than 10% in the 8–13 µm wavelength region, with a minimum of 1.37%. A statistical analysis of the distribution of materials and geometrical parameters that make up the solar absorbers is presented.

## Introduction

In the pursuit of renewable and green energy sources, the sun provides an enormous amount of energy waiting to be harvested in a meaningful way. Perfect solar absorbers play an important role in solar energy harvesting by converting photons into thermal energy [1, 2]. Using perfect solar absorbers allows all of the absorbed energy to be used in the conversion process. An ideal solar energy absorber should have two main properties. First, it should absorb all wavelengths of electromagnetic radiation that reach the Earth, and second it should not radiate that absorbed energy away as heat. This process allows the absorbed solar energy to be completely converted to other forms of energy for practical everyday use. The proposed materials in this work can also be used as perfect absorbers in the visible region.

One way to produce such solar absorbers is through metamaterials. Since their introduction by Pendry et al. [3], metamaterials have been used for numerous applications, such as light absorbers [4–15], cloaking devices [16, 17], and nonlinear optics [18, 19]. By carefully designing the subwavelength geometrical properties of metamaterials, their optical properties can be manipulated for specific purposes. The design process is usually performed using knowledge from previous research and the intuition of the researcher. This can be an arduous process for more complex designs. Recently, artificial intelligence (AI) has been used to help find solutions to complex problems and uncover underlying relationships between the design parameter space and the optical properties in the field of nanophotonics [20]. Neural networks have been used for research in optics recently to design nanophotonic structures [21–27] and chiral metamaterials [28], predict the optical properties of structures [29], and for signal processing [30].

The deep Q-network (DQN), a reinforcement learning algorithm is a powerful tool that can be used to optimize solutions for a problem [29, 31–35] by acting as an intelligent search. Through exploration, the network takes actions and receives feedback, allowing it to learn about the parameter space and make intelligent choices. This method and its benchmarks have been explained in more detail in a number of articles [32, 33, 36, 37]. In contrast to other deep learning, reinforcement learning does not learn the hidden nonlinear relationships in a predetermined dataset but uses rewards and punishments in order to maximize a given reward. To start with there is no dataset, but through exploration and exploitation of the data space by an agent, it learns how to traverse the space and make good decisions to maximize its long-term reward.

## Methods

### Structure

**Fig. 1:**
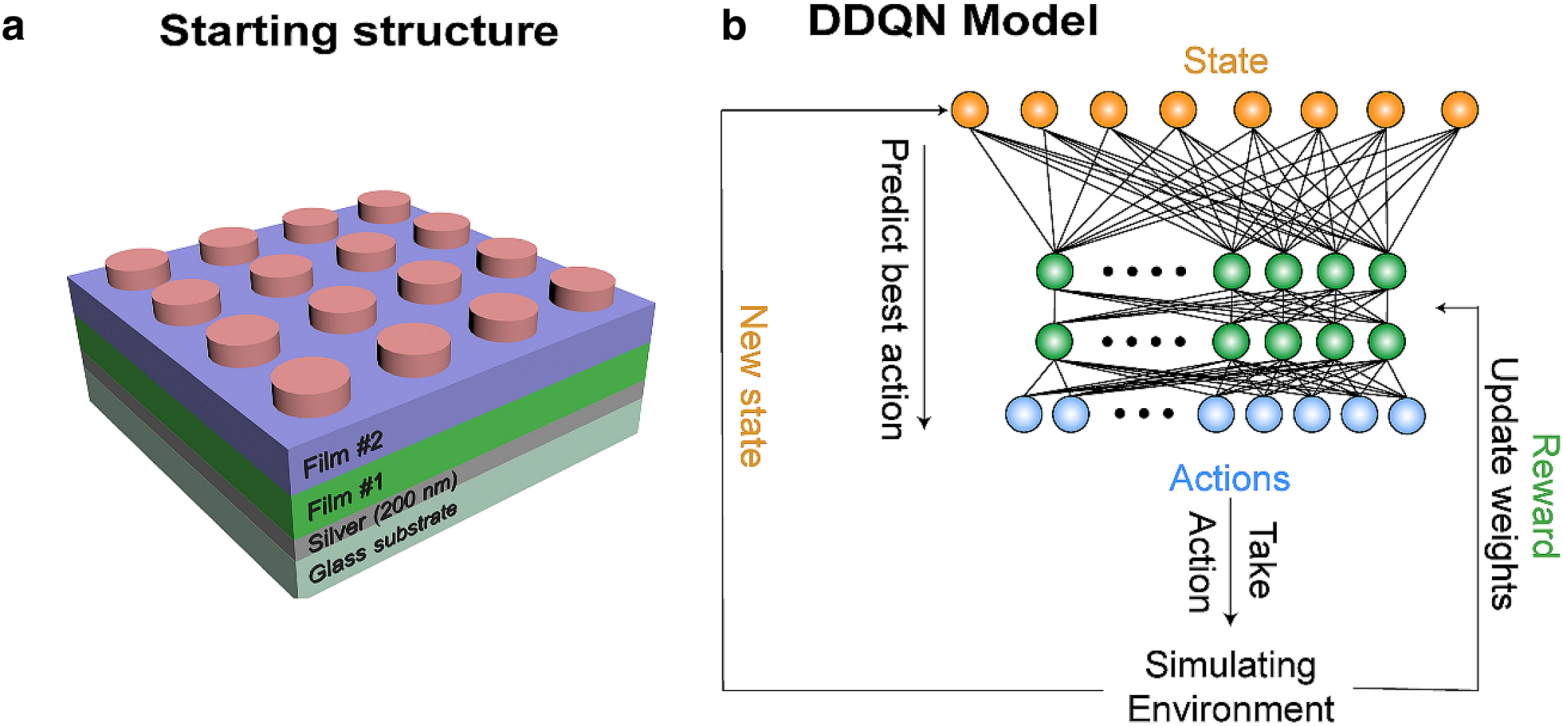
**a** A schematic of the structure for the DDQN model to optimize and **b** the algorithm flowchart of the DDQN model

A schematic of the initial structure is shown in Fig. 1a. It is composed of an array of nanocylinders on top of a silver back reflector and 2 film layers, all on a glass substrate. This structure was inspired by our previous experimental experience. The starting point can be chosen arbitrarily, but a well-educated guess can help to reach final results faster. The cylinders assure that the final structure will be polarization independent, while the bottom layer is a 200 nm silver back reflector, as is common in the design of many perfect absorbers. Lots of research has been published based on this type of structure with a variety of different number of layers and shapes [6, 38, 39]. Here, the geometrical parameters and materials of the nanocylinders and layers are chosen by the DQN.

### Deep Q-network (DQN)

The DQN was originally introduced as an AI agent that can play videogames at a level that can rival human players [33, 40]. The DQN has been able to complete different games with the same algorithm. In videogames, each new screen is a new state where the agent can take an action, since there are so many possible states and actions, it is impossible to explore them all, or to use conventional algorithms to solve the game. A DQN starts by exploring a game and gradually learning the mechanics of it, the more the agent plays this game, the more it learns and is able to achieve higher scores. In this work, the DQN will learn the connection between the change of geometrical properties and their effect on final results through full wave FDTD simulations, and then use that knowledge to design structures that produce the optical responses that we desire. First, the environment is set up, this includes the initial structure design and the simulation environment, second, the actions that the agent can take to change the structure are decided and finally, the reward system is defined. The DQN algorithm that connects all these parts together is shown in Fig. 1b.

The decision of which action to take in a given state is decided by a neural network that is updated based on what it has learned. To improve the performance of the DQN, an auxiliary model is used alongside. This network is used to select the action for the agent to take, while the main DQN network is used to predict the Q-value of the state-action pair. This prevents the overestimation that is a problem in general DQN. At each iteration, two models are trained, and the weights of the target model are gained from the combination of the main model weights and the target model weights. This method helped the overestimation caused by using just one model. The auxiliary network is updated periodically with the parameters of the DQN. Since there are two networks working together, this is known as a double deep Q-network (DDQN) [41]. The rule for how an action is chosen is called the policy and the set of action, state, and policy form a Markov decision process (MDP). An MDP means that in a given state, the policy that is used to decide which action to take is based on the previous rewards gained from previous states and actions. The full details of this model and a pictorial comparison of the two q-network models is given in our previous work [24, 42]. Each neural network has 3 hidden layers with 12 neurons.

### State

The state, which is an array of the materials and geometrical properties of the structure, its variations, and limits are defined as follows:

- Cylinder material: 1 of 13 materials (Table 1).

**Table 1:** The materials available to be used for the films and nanocylinders

ID#	Material	ID#	Material
1	ZnS	8	InAs
2	TiO_2_	9	InP
3	PDMS	10	Ge
4	Al_2_O_3_	11	Si
5	ZnO	12	Si_3_N_4_
6	TiN	13	SiO_2_
7	GaAs
- Film #1 material: 1 of 13 materials (Table 1).
- Film #2 material: 1 of 13 materials (Table 1).
- Cylinder diameter: 0–200 nm, step size: 10 nm.
- Cylinder thickness: 0–200 nm, step size: 10 nm.
- Film #1 thickness: 0–2000 nm, step size: 10 nm.
- Film #2 thickness: 0–2000 nm, step size: 10 nm.
- Gap between cylinders: 50–200 nm, step size: 10 nm.

The total number of possible states is therefore, 13 × 13 × 13 × 20 × 20 × 200 × 200 × 15 = 527,280,000,000. Manually searching all of these states is impossible, but the DDQN can produce desirable results in a reasonable time. This will be discussed in more detail in the results section. It should be noted that the number of materials and geometrical properties can be chosen arbitrarily. Choosing a larger range of values could lead to better results but would take longer to train and converge. This is limited only by the available resources.

The initial state is defined as the central value of each parameter, i.e. cylinder, film #1 and film #2 materials: material 7 (GaAs), cylinder diameter: 100 nm, cylinder thickness: 100 nm, film #1 and film #2 thicknesses: 1000 nm and the spacing between cylinders: 100 nm

### Actions

The actions available to the agent to change the geometrical properties of the design at each step are shown in Table 2. As with all numerical methods, the parameter space is continuous, so it is discretized into smaller steps, as defined in “State” section. A step size of 10 nm for the cylinder diameter is chosen as it was deemed an appropriate accuracy through testing. At each update, the model learns from its previous states, actions and rewards and decides the best action to take next.

**Table 2:** Definitions of the actions available to the agent

Action no.	Action definition
0	Decrease the spacing between cylinders by 10 nm.
1	Increase the spacing between cylinders by 10 nm.
2	Decrease the height of the cylinder by 10 nm.
3	Increase the height of the cylinder by 10 nm.
4	Decrease the diameter of the cylinders by 10 nm.
5	Increase the diameter of the cylinders by 10 nm.
6	Decrease the thickness of film #1 by 10 nm.
7	Increase the thickness of film #1 by 10 nm.
8	Decrease the thickness of film #2 by 10 nm.
9	Increase the thickness of film #2 by 10 nm.
10	Decrease the material ID of cylinders by 1.
11	Increase the material ID of cylinders by 1.
12	Decrease the material ID of the film #1 by 1.
13	Increase the material ID of the film #1 by 1.
14	Decrease the material ID of the film #2 by 1.
15	Increase the material ID of the film #2 by 1.

### Reward system

The reward system gives feedback to the agent by giving information about how well it is learning. This is where the problem is set up to find a perfect solar absorber. A perfect solar absorber should have perfect absorption in the visible regime (350 to 800 nm) to absorb all the solar energy, while having minimum absorption in the mid-IR range of 8 µm to 13 µm to not radiate it back out as heat. An area under the curve (AUC) value for each absorption spectrum was calculated in each region and the reward function was designed as follows:

1 $$reward = 200 + absorption AUC\left( {350 - 800 nm} \right)\% - absorption AUC\left( {8 - 13 \mu m} \right)\%$$

200 is added to the reward to make sure that it remains positive. An ideal structure will gain a reward of 300, while the worst structure gets a reward of 100 (since the AUC ranges from 0 to 100%). The absorption over each range of wavelengths was calculated with power monitors for reflection (R) and transmission (T), taking the absorption (A) to be A = 1−R−T.

## Results and discussion

**Fig. 2:**
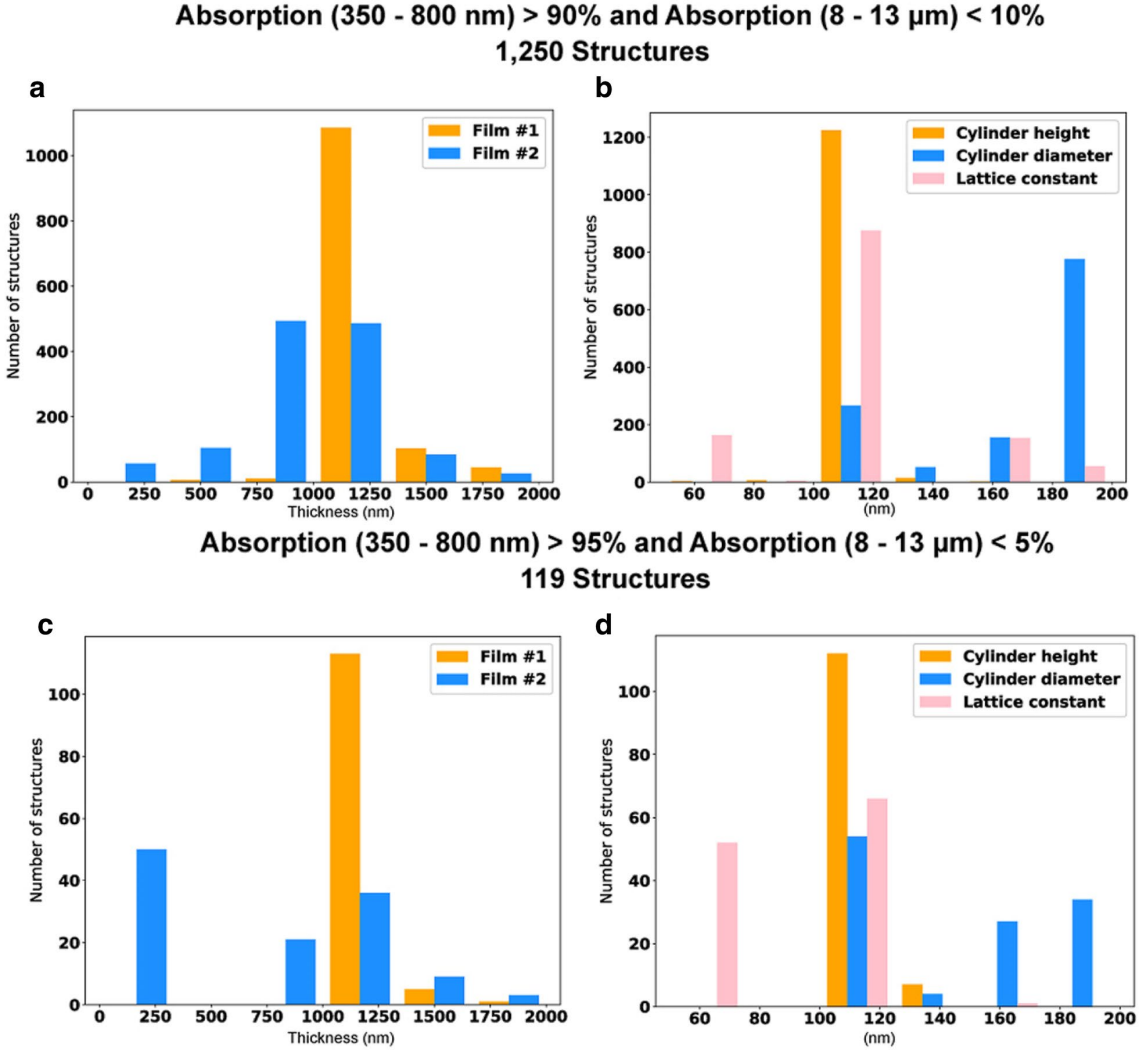
**a, c** Histograms of film #1 and film #2 thickness distributions, and **b, d** cylinder height, diameter, and lattice constant distributions for two criteria. **a, c** show the distributions for the structures with an AUC higher than 90% from 350–800 nm and lower than 10% from 8–13 μm (1,250 structures). **b, d** show distributions structures with an AUC higher than 95% from 350–800 nm and an AUC lower than 5% from 8–13 μm (119 structures)

The simulations were performed using a computer with a 3.40 GHz 16-core processor, 64 GB of RAM, and an NVIDIA RTX 2070 GPU with 8 GB DDR6 RAM. At each step, the DDQN code was run in Python for the AI calculations and connected to Lumerical, a commercial FDTD solver, to evaluate its predictions. All material data was taken from the inbuild database. This method requires a PC with both a strong CPU for the FDTD simulations and GPU for the neural network calculations. With this setup it took around 1 month, where around 35,000 steps were taken. In the process of uncovering the best structures, the DDQN finds many similar solutions with small differences in their rewards. This means that many structures with acceptable results and different geometrical properties are discovered, allowing for a statistical analysis. A situation called backflipping occurred, which means that the model is stuck in a single configuration. This issue was fixed by tuning the hyperparameters. Figure 2 shows histograms of the distributions of different geometrical properties for film #1, film #2, the cylinder and the lattice constant. These graphs are prepared for two different categories. The first shows the top 10% of structures, which have an AUC of higher than 90% in the 350 to 800 nm wavelength region and lower than 10% in the 8 µm to 13 µm region. The second displays the top 5% of structures, which have an AUC higher than 95% in the 350 to 800 nm wavelength region and lower than 5% in the 8 µm to 13 µm region. The DDQN produced 119 structures with these properties. The DDQN uncovered 1250 structures with these properties. shows histograms of the distributions of different geometrical properties for film #1, film #2, the cylinder and the lattice constant. These graphs are prepared for two different categories. The first shows the top 10% of structures, which have an AUC of higher than 90% in the 350 to 800 nm wavelength region and lower than 10% in the 8 µm to 13 µm region. The second displays the top 5% of structures, which have an AUC higher than 95% in the 350 to 800 nm wavelength region and lower than 5% in the 8 µm to 13 µm region. The DDQN produced 119 structures with these properties. The DDQN uncovered 1250 structures with these properties.

**Fig. 3:**
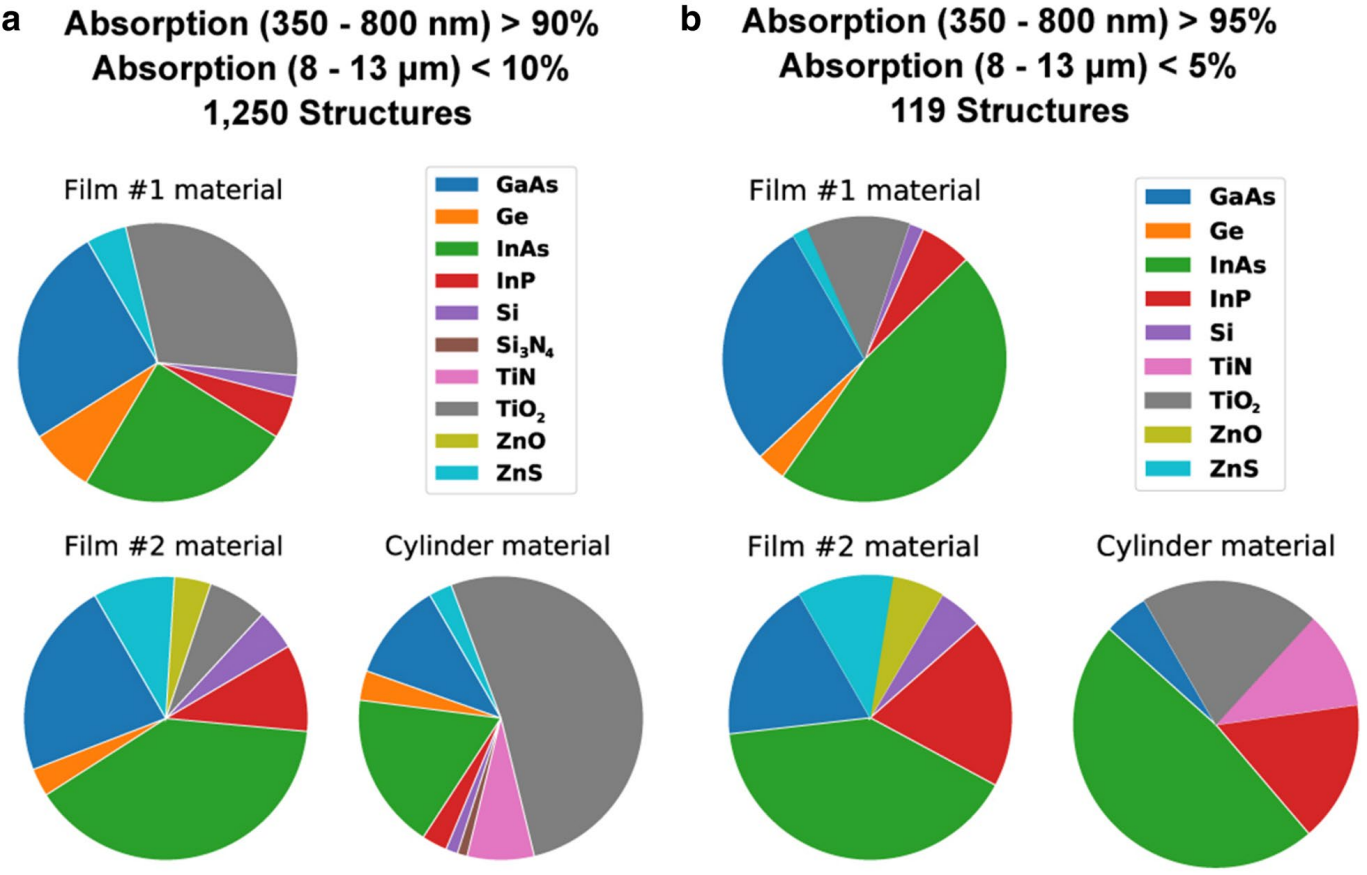
Pie charts of (**a**) the material distributions of film #1, film# 2 and the cylinder for structures with an AUC higher than 90% from 350 to 800 nm and an AUC lower than 10% from 8 to 13 μm (1250 structures) and (**b**) for the structures with an AUC higher than 95% from 350 to 800 nm and an AUC lower than 5% from 8 to 13 μm (119 structures)

**Fig. 4:**
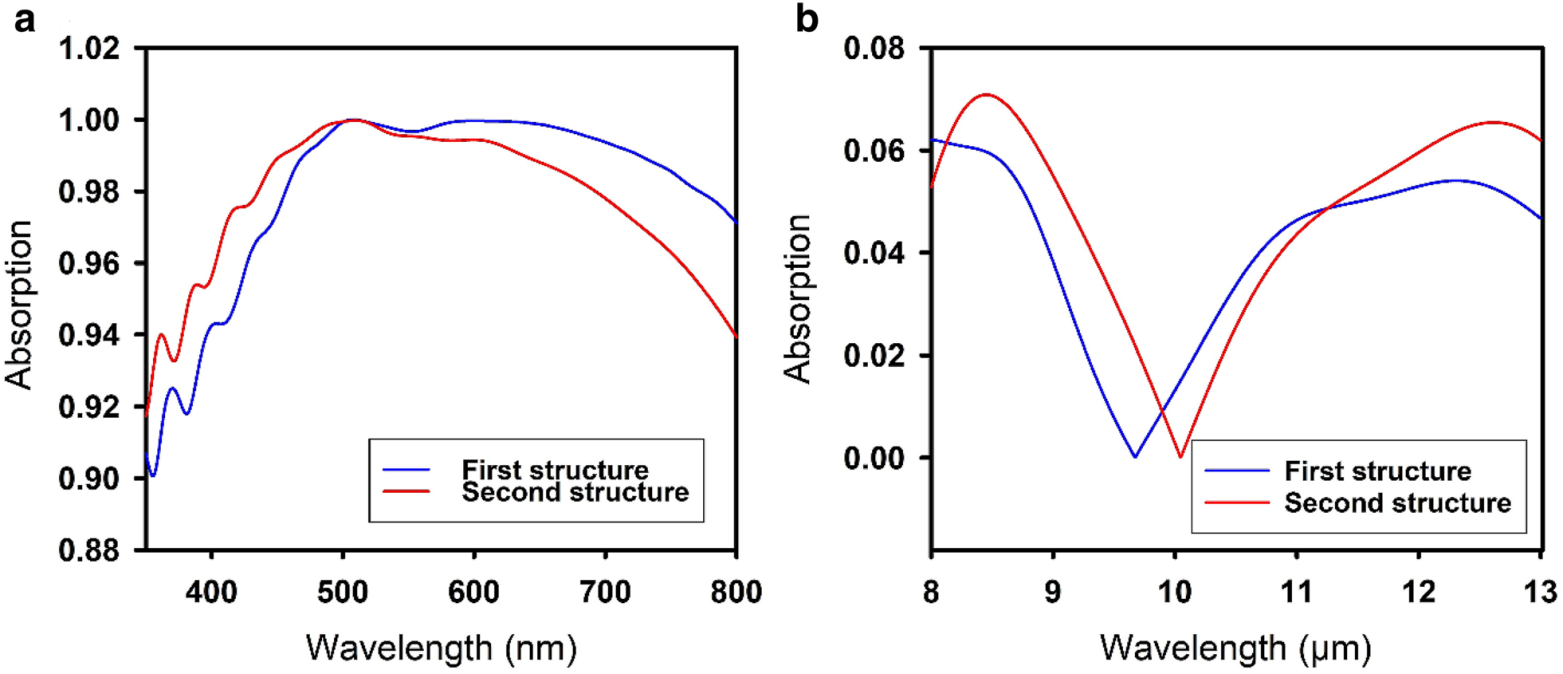
Absorption curves of the top two performing structures from Table 3 for (**a**) 350–800 nm and (**b**) 8–13 µm

The distributions of the material choices for film #1, film #2 and the cylinder are displayed in the pie charts in Fig. 3 for the same categories as previously described. These plots reflect which materials should be chosen to obtain perfect solar absorbers. Table 3 shows the materials and geometric parameters of the top 8 performing structures discovered by the DDQN, with the absorption curves of the top 2 shown in Fig. 4. In comparison with human findings, A. Al-Rjoub et al. [43] reported almost identical theoretical and experimental results of a 95.2% and 9.8% absorption in the first and second wavelength regions.

**Table 3:** Some of highest efficiency structures by the DDQN

Film #1 mat.	Film #2 mat.	Film #1 height (nm)	Film #2 height (nm)	Cylinder mat.	Cylinder height (nm)	Cylinder diameter (nm)	Lattice spacing (nm)	Abs. AUC (350 – 800 nm) %	Abs. AUC (8 – 13 µm) %
Ge	GaAs	1120	40	InAs	100	100	50	97.61	1.37
InAs	InP	1000	240	InAs	100	100	50	97.32	1.50
GaAs	InP	1120	40	InAs	100	100	50	97.05	2.48
InAs	GaAs	1120	40	InAs	100	100	50	96.75	2.56
InAs	GaAs	1110	40	InAs	100	100	50	96.74	2.67
GaAs	GaAs	1120	40	InAs	100	100	50	96.57	2.58
InAs	ZnO	1300	290	TiN	140	100	120	96.54	2.83
InAs	InAs	1000	160	InAs	100	100	50	96.53	2.85

## Conclusions

A DDQN was used to design structures to be used as solar perfect absorbers in a parameter space that allows for 527 billion possible designs. Using a variety of materials, it was able to produce around 1250 perfect solar absorbers in around 35,000 steps. Each structure has an AUC higher than 90% in the visible region with low absorption in the 8 µm to 13 µm region. A statistical analysis was produced to help readers choose suitable geometrical properties and materials based on their fabrication limitations to design a perfect solar absorber.

## Data Availability

The datasets generated and/or analyzed during the current study are not publicly available due to the funding agency’s regulations but are available from the corresponding author on reasonable request.
